# Emergency Care for Women Irregular Migrants Who Arrive in Spain by Small Boat: A Qualitative Study

**DOI:** 10.3390/ijerph16183287

**Published:** 2019-09-06

**Authors:** Esperanza López-Domene, José Granero-Molina, Cayetano Fernández-Sola, José Manuel Hernández-Padilla, María del Mar López-Rodríguez, Isabel María Fernández-Medina, Maria Dolores Guerra-Martín, María del Mar Jiménez-Lasserrrotte

**Affiliations:** 1 Cruz Roja Española, 04002 Almería, Spain; 2 Department of Nursing, Physiotherapy and Medicine, University of Almeria, 04120 Almería, Spain; 3 Faculty of Health Sciences, Universidad Autónoma de Chile, Temuco 4780000, Chile; 4 School of Health and Education, Middlesex University, London NW4 4BT, UK; 5 Faculty of Nursing, Physiotherapy and Podiatry, University of Sevilla, 41009 Sevilla, Spain

**Keywords:** migrant, women, vulnerable, public health, European union

## Abstract

Background: this study aimed to describe and understand the experiences and health needs of women irregular migrants during emergency care provision upon arrival in Spain by small boat. Methods: a qualitative study based on Gadamer’s phenomenology was used. The data collection included 13 in-depth interviews with women irregular migrants and 10 in-depth interviews with key informants. The study took place in the Spanish Red Cross’ facilities between February 2017 and April 2018. Results: two main themes emerged from the data analysis: the need for emergency care focused on women irregular migrants with the sub-themes ‘Women irregular migrants as objects of sexual exploitation’ and ‘The mother-child dyad as the axis in human trafficking’; and developing an emergency care gender policy for women irregular migrants, with the subthemes ‘Healthcare in a police-controlled setting: detecting weaknesses’ and ‘Promoting screening and safety protocols focused on women irregular migrants’. Conclusions: women irregular migrants who arrive in Spain by small boat have specific needs and healthcare problems. Due to strict safety conditions during emergency care provision, rape and human trafficking can go unnoticed. Implications: interdisciplinary care protocols and new health policies that have a gender perspective are needed to improve the emergency care provided to women irregular migrants.

## 1. Introduction

The European Union (EU) receives a third of the global migrant population, many of whom are irregular migrants (IMs) [1,2]. Irregular migrants (IMs) are not authorized to enter or stay in the country to which they migrate, as they do not have legal permission, documentation or refugee status. IMs arrive in the EU after having fled wars [3], sexual violence, racial, political or religious conflicts or extreme poverty [4,5]. IMs that arrive in the EU come from the Middle East, North Africa and Sub-Saharan Africa [6]. In 2015–2016, more than a million IMs arrived in the EU by sea, crossing the Mediterranean Sea on a small boat which resulted in the death and disappearance of 6000 migrants [7,8]. The small boats are semi-rigid inflatable vessels with two engines and no keel (Zodiac-style boats), about 5–10 meters in length, which can fit between 40–50 people along with fuel tanks. Thousands of Maghrebis and sub-Saharan Africans arrive in Spain by small boat, risking their lives on the high seas without food or water [9,10]. IMs are a vulnerable population with health risks that require specific attention. Institutions such as the Red Cross take on the responsibility of rescuing and providing emergency care. The Maritime Rescue Team intercept the small boats offshore and alert the Red Cross Emergency Response Team (doctor, nurses, volunteers, cultural mediators [11]. Migrants may experience a number of health issues caused by the living conditions faced during the migratory journey, and overcrowding could also favour the occurrence of outbreaks [12]. Emergency care provided to IMs includes triage, first aid, food and water provision [2], screening for transmissible diseases diagnosis, treatment, and the identification of survivors and the deceased [13,14,15]. Although the majority of IMs who arrive in Europe by small boat are men, the number of women and children is on the rise [6,16]. Women irregular migrants (WIMs) are a vulnerable population [17,18]; when making the journey they are faced with physical and psychological problems including violence, sexual exploitation and undesired pregnancies [19,20]. WIMs who arrive in Spain by small boat need care for their sexual and reproductive health as well as protection from human trafficking [21]. The arrival of IMs in the EU by small boat is a topic that has been researched on an epidemiological and political level and in terms of needs and resources [22,23,24,25] but little is known about the experiences of WIMs when receiving emergency care. Additional research is needed, from a service delivery perspective, about how to provide better care to WIMs. Following the framework developed by Zimmerman, et al. [26], this study aimed to describe and understand the experiences of WIMs during emergency care provision upon arrival in Spain by small boat.

## 2. Materials and Methods 

### 2.1. Study Design

A qualitative descriptive approach based on H.G. Gadamer’s hermeneutic phenomenology was adopted [27]. According to Gadamer, understanding the meaning that people assign to their experiences is fundamental to practice. Hermeneutic phenomenology allows us to interpret experiences, to understand a text (or transcription) and also implies the pre-understanding of the researcher, in a fusion of horizons that brings about new knowledge. Experiences and emergency care for WIMs arriving in small boat is a phenomenon that can be explored from these perspectives. We have used the Consolidated Criteria for Reporting Qualitative Research (COREQ) [28] as our guideline for reporting of this study.

### 2.2. Setting

The study took place in the Spanish Red Cross facilities between May 2017 and June 2018.

### 2.3. Participants

Purposive sampling was used to recruit both groups of interviewees, both WIMs and key informants (KI). The inclusion criteria were: to consent to participating in the study (both groups), to have received emergency care after crossing the Mediterranean Sea in a small boat between 2015–2017 (WIMs), or to have a minimum of one year of experience in providing emergency care to WIMs (KIs). The exclusion criteria for both groups was to refuse to participate in the study and to be <18 years old. The initial list consisted of 30 WIMs residing in suburban or agricultural settlements or in Red Cross Humanitarian Settlement Centres. After making contact, 22 WIMs satisfied the inclusion criteria and accepted to participate in the study. Ultimately, only 13 WIMs were included (five left the study for work reasons, one due to illness and three moved settlement). All selected KIs agreed to participate in the study (Table 1). The WIMs’ interviews were conducted in French, with the help of cultural mediators; the KIs’ interviews were conducted in Spanish. 

### 2.4. Data Generation

In-depth interviews were carried out with 13 WIMs (with an average duration of 18 min) and with 10 KIs (with an average duration of 34 min). The average time between arrival in Spain by small boat and the in-depth interview was 39.2 days. In-depth interviews were carried out by two researchers, accompanied by cultural mediators who translated the questions and responses into the participants’ language (French), and who had received relevant training and practised the interview protocol (Table 2). All participants’ responses were recorded, transcribed, returned to participants to be checked, and incorporated in a hermeneutic unit that was later analysed using the software Atlas-ti 7.0 (Thomas Muhr, Berlin, Germany). Upon reaching data saturation, data collection ceased. 

### 2.5. Data Analysis

Valerie Fleming’s stages were used [29]. Firstly, the researchers gave an affirmative response to the question: *Can one study the experiences of WIMs who arrive by small boat using hermeneutic phenomenology?* Secondly, the researchers reflected upon their pre-understanding of the phenomenon. The third step was to understand the phenomenon through speaking with the participants. The fourth step aimed to understand the phenomenon through text analysis. After reading the transcriptions, the participants’ experiences were re-examined and analysed. Three members of the research team codified the data independently and compared their interpretations through an inductive process of collecting quotations, units of meaning, sub-themes or themes. In the fifth stage, the reliability and rigour of the qualitative data was established. To increase reliability, a clear description of the context, data collection and data analysis was provided. Triangulation between researchers was performed, as three researchers analysed the data separately before coming to an agreement. To guarantee conformability, each transcription was reviewed by the participants, who identified the content and verified the results.

### 2.6. Ethical Considerations

WIMs’ interviews were conducted with the help of cultural mediators, in French, which was spoken by all of the participants, and their understanding of the language was confirmed. Throughout the interview, any doubts were clarified and any necessary explanations were given. After transcription, the information gathered during the interview was individually checked with the participants, in order to ensure that the events described in their statements had been correctly interpreted and accurately reflect the their experience. All subjects gave their informed consent for inclusion before they participated in the study. Approval was gained from the Spanish Red Cross Ethics and Research Committee (CR-1501). 

## 3. Results

The definitive sample comprised 13 WIMs from eight countries with an average age of 28.6 years (standard deviation (SD) = ± 6.3); and 10 key informants with an average age of 36.7 years (SD = ± 11.1). Two themes and four subthemes (with their units of meaning) (Table 3) allow for the experiences of WIMs during emergency care provision upon arrival in Spain by small boat to be understood. 

### 3.1. The Need for Emergency Care Focused on Women Irregular Migrants (WIMs)

This theme describes the specific risks of WIMs on this dangerous voyage. Violence, rape, unwanted pregnancy and being used by trafficking networks, in the case of child immigration, all justify the need to implement specific emergency care protocols that are focused on women. 

#### 3.1.1. WIMs as Objects of Sexual Exploitation

All of the participants agree that crossing the Mediterranean Sea is the last phase of a dangerous journey across many countries. The conditions regarding hygiene, food, health and violence are abysmal. WIMs board small, crowded boats without water or food when pregnant or with a small child. Emergency care must be focused on their safety and needs.

*“Upon disembarking, a woman gave us hot chocolate, a sandwich, a shower and dry clothes…after that, they took me to the hospital”.*

(WIM-5)

WIMs mostly come from sub-Saharan Africa, suffer extreme violence during the journey and arrive feeling exhausted and fearful. After their rescue, nurses, volunteers and cultural mediators carry out a physical assessment and provide emergency care. Emergency care deals with hypothermia, dehydration, malnutrition, burns and trauma. However, WIMs (mostly from sub-Saharan Africa) hide symptoms of abuse, rape and unwanted pregnancy.

*“A young WIM wanted to know if she was still a virgin, after they had tried to rape her on the journey”.*

(KI-5)

Although their goal is to start a new life in Europe, each WIM has their own story. Maghrebi women usually travel with their husband and/or children, so sexual exploitation is rare. Sub-Saharan WIMs, such as many Nigerian women, are usually adolescents who come from poor environments where structural violence is commonplace in their place of origin. They are especially susceptible, being urged by their families or taken advantage of by other women, and suffer more on the difficult journey and are likely candidates for sexual exploitation. One participant described it in this way.

*“When my brother paid (the trafficking network) they picked me up in a parking lot... when I got in the car I saw two young skinny girls lying in the boot of the car”.*

(WIM13)

After emergency care has been provided, an initial interview is conducted in order to identify network trafficking. For the majority of our informants it is a complicated task. As one of the WIMs confirms, they are terrified and confused after the journey.

*“You arrive feeling tired, sleepy, I couldn’t talk. The sea is terrifying, you are not aware of what is ahead”.*

(WIM-1)

The participants agree that sexual assault is constant throughout the journey. WIMs are forced into sexual contact by traffickers and police in transit countries. Their bodies are used by trafficking networks as a way of payment to cross borders.

*“They come and rape you for days and when it interests them, they leave you there, bleeding… and you have to carry on with the journey as best as you can”.*

(WIM-3)

To identify WIMs who have been victims of human trafficking and prostitution, emergency care provision includes a physical assessment that looks for marks on their clothes or bodies, scars, tattoos or bruising. According to nurses and cultural mediators, WIMs do not want to talk about this matter and they hide it out of fear and shame and because they fear violent punishments. A WIM mentioned:

*“I wanted to escape and they tied me up to a pole for three days in the sun… they burned chest with boiling water”.*

(WIM-8)

WIMs cannot access health services during the journey so the emergency care received upon arrival in Spain must include a gynaecological assessment and an evaluation of the WIMs’ reproductive history, pregnancies, births and miscarriages. As a nurse states, WIMs require a pregnancy test, a foetal examination and a diagnosis of infection or genital mutilation.

*“A Sub-Saharan woman asked us for a pregnancy test and cried tears of joy upon receiving the negative result. She had been in a car for five days and had been raped by three men”.*

(KI-9)

The trafficking networks decide if they will arrive in Europe pregnant or whether they will take a form of contraception. Some informants told us that WIMs (mostly from sub-Saharan Africa) are deprived of their sexual and reproductive autonomy and are obliged to have illegal abortions in Morocco or Algeria at a high risk for their health.

*“The network decides if they get pregnant and when or if they will have a child. Sometimes they force them to have abortions in advanced pregnancies”.*

(KI-6)

Female genital mutilation is a common practice in sub-Saharan Africa and is carried out by healers on WIMs when they are children or even adults. The percentage of women who undergo female genital mutilation in some of these countries ranges from about 56% in Gambia, 17% in Nigeria, 13% in Burkina Faso and Senegal and 10% in Ivory Coast [30]. Although many countries now penalise this practice for being a violation of human rights and a health risk, the prevalence continues to be high [31]. For all participants it is a challenge to identify these practices. WIMs do not want to speak about the matter so emergency care should include a gynaecological assessment and a transfer to hospital if necessary.

*“They carried out genital mutilation on me when I was 8. When I arrived in Spain they took me to the hospital … Everything was OK”.*

(WIM-3)

Trafficking networks coerce WIMs into prostitution through witchcraft, voodoo and magic. Emergency care must identify these practices and help the women. According to a key informant, the physical assessment should look out for bleeding, scars, marks and even charms that are used as a way of protecting themselves, their children or their families.

*“The trafficking networks use voodoo because Africans have strong beliefs; they tie a rope around their waists and carry a little bag of hair…to protect themselves from ‘juju’”.*

(KI-5)

Emergency care teams observe, listen and gather information about the WIMs who may be victims of human trafficking. The WIMs are distrustful, they hide information and they do not report abuse or ask for protection. The madame does not travel in the small boat, but there may be a female “controller” who presides over the rest in the boat, who also is a victim. This is how one informant describes it:

*“Sometimes I observe fear, and notice WIMs’ submission when they make the journey with a woman who controls the whole group! But we cannot make any mistakes when judging who it is... because she is just another victim of the network herself!”.*

(KI-3)

The poor state of the boat itself, the heavy load and meteorological factors can lead to the boat sinking and the migrants dying. This is how a WIM describes it.

*“The waves hit the boat and it moved a lot. We were very nervous and some people, (including children) fell in the water and they didn’t know how to swim … Women took the corpses of the children who had drowned, shook them, told them to wake up, cried and shouted …”.*

(WIM-9)

#### 3.1.2. The Mother-Child Dyad as the Axis in Human Trafficking

The journey husband (a member of the network) controls the WIMs’ sexual lives and allows them to be raped and impregnated on the journey. Before even embarking on the small boat, the WIMs’ documents are taken away and they are given a child (generally their own, although sometimes this is not the case). The emergency care team must observe the mother-child relationship; if children have a bond with her or another woman, if the mother watches over the child or if she is indifferent, and if she feeds the child or they have to be fed by a nurse.

*“We left the child with the WIM who had brought them, observed how they moved, to whom they got close and whether the WIM consoled them”.*

(KI-2)

If the WIMs come as a family, the parents are questioned separately over personal details about themselves and the child (growth, illness). The cultural mediators are key in identifying people trafficking, despite it being the police’s responsibility.

*“They asked for my name, nationality, my child’s and my husband’s names … They also asked me about my family several times … They even asked my husband afterwards”.*

(WIM-11)

After disembarking, the children go straight to the infirmary for an assessment along with the WIM who has accompanied them. The team observes the mother-child relationship: the similarity of physical features; whether they look at each other in the eye; and whether the WIM knows how to dress or calm the child. As a nurse states, the trafficking networks assign or take children away from WIMs by threatening them. Many of the WIMs will be prostituted in Europe and they do not want them to feed so that their breasts do not worsen aesthetically.

*“The networks prevent WIMs from feeding their children. They don’t want them to be damaged because they will later be prostituted”*

(KI-8)

The emergency care team informs the WIMs that there will be DNA testing to know if they are family, the WIMs can get scared and admit that they are not their mothers. Or as one nurse says, a WIM and her son might have been separated since birth in order to sexually exploit the mother. All these problems can be detected by the emergency care team.

*“The woman took the baby, gave it a push and said that is your Mum … and the child said no, mami, no …!”*

(KI-10)

In the case of some WIMs, the families push underage girls to emigrate to send money home. People trafficking networks oblige the children to say that they are of legal age in order to exploit them sexually. In recent years, the recruitment age has decreased and many minors are sexually exploited. Women are still afraid, WIMs do not want to discuss the topic because they do not want to implicate their families.

*“My parents want a better life for me. They want me to make the journey to help them … They don’t know how dangerous it is. I don’t want to talk about this!”*

(WIM-12)

### 3.2. Developing a Gender Policy for WIMs in Emergency Care

This theme focuses on the public health issues regarding care and treatment of WIMs. Problems and activities that should be incorporated into first emergency care protocols to IMs are defined.

#### 3.2.1. Healthcare in a Police-Controlled Setting: Detecting Weakness

Many WIMs suffer from poverty and inequality in their countries of origin. They migrate in search of access to education, as well as to escape violence and social and gender discrimination. After the journey through the desert and by small boat, they receive their first emergency care and are detained by the police. They had imagined an idealistic life in Europe, but their expectations drastically change once they are faced with their new reality.

*“I wanted to come to Europe for a better life, but they locked us up... we haven’t done anything, why would they do that? We are not criminals”*

(WIM-6)

WIMs do not have permission to enter the country and are arrested by the police upon arrival. Emergency care is limited by the police identification efforts in a context in which safety prevails over healthcare delivery. In the nursing section, care is provided for IMs of different sex, age, language and culture. For the majority of participants, it is fast service for physical problems; but there is no time to explore psychological, sexual or gender-related problems.

*“There is no intimacy nor time to ask and listen. In five minutes, they aren’t going to tell you anything! It is very difficult to detect human trafficking in these women”*

(KI-7)

Cultural mediators are needed, as they speak the WIMs’ language, facilitating communication and trust during emergency care. WIMs feel safe when a fellow African speaks to them (in English, French, Arabic …), because he/she is aware of their customs, suffering, experiences and problems. After being identified and provided with emergency care, the WIMs are detained in police units for days. For the emergency care team, this is a good moment to explore their life history: sexual abuse, trafficking, stigma, hopes for the future. However, as one WIM says, it is not an appropriate time to talk about trafficking upon arrival.

*“Doctor asks, police asks…I couldn’t speak, I was tired, water came into small boat … all night”.*

(WIM-7)

#### 3.2.2. Promoting Screening and Safety Protocols Focused on WIMs

WIMs need emergency care with a multidisciplinary and multicultural approach; to recognise their vulnerability, health problems (HIV, sexually-transmitted diseases) and ensure their safety. The nurses insist on the need for gynaecological assessments (signs of pregnancy, infection, mutilation), blood tests (serology), but also sensitivity and respect. WIMs live through difficult experiences on the journey so their emotional state is fragile and unpredictable. They require affection, understanding and knowing what is their final destination. They come across as distrustful and depressed. All participants explicitly state that emergency care must incorporate mental health screening because WIMs arrive stretched to the limit with nothing more than their faith to console them. This is what a WIM remembers.

*“The journey was terrible, indescribable … I knew that small boat was a matter of life or death. I left everything in the hands of Allah”.*

(WIM-6)

WIMs do not have documents and they are detained in cells along with the children. The mediators explain their rights to them, how to seek asylum, and whether they will be freed or sent to an internment facility. According to WIMs, it is a sad and traumatic experience.

*“In a cell, they don’t speak Arabic, I don’t know if they will return me to Morocco … we are not criminals!”.*

(WIM-7)

After the provision of emergency care, a risk report is completed and suspicions over trafficking, kinship or age are highlighted. Some participants agree that the objectivity of these reports must be improved, but we need time to gain their trust and to protect WIMs.

*“We need protocols adapted to WIMs and children. Knowing what to do, when and how, it would give us a safety and a higher guarantee of success”.*

(KI-1)

Emergency care for WIMs needs joint protocols between the Red Cross and the police. It is unanimously agreed that there is a lack of organisation, well-defined roles, when the police should act and when the nurse should act, female police officers or female mediators. As one team leader states, without these measures, WIMs will continue to be unprotected.

*“If we take one step, the trafficking networks takes three. First the women are alone, then with children, then pregnant”.*

(KI-3)

WIMs have debts that they need to pay back to the trafficking network, and they fear that their families will be victims of violence. They are being watched and feel guilt, shame and fear. The trafficking network used children to control the WIMs and this worries them.

*“Who protects my baby? Who protects my family? I will say no more!”.*

(WIM-4)

## 4. Discussion

This study aimed to describe and understand the experiences and health needs of WIMs during emergency care provision upon arrival in Spain by small boat. The EU receives mixed immigration with an increasing number of women and children [6,16]. The relationship between immigration policies and healthcare results requires the development of new strategies in emergency care for WIMs [17,32]. 

As in other studies [33], for WIMs the category of being ‘unauthorised’ or ‘irregular’ forces them to remain hidden and also exposes them to greater danger and risk. Our study confirms that when women and children cross borders and they use illegal routes, they are generally afraid to give too much information [34]. Many WIMs have been victims of violence, that come with physical, sexual and mental trauma [21]. Corresponding with other studies, a high morbidity rate, low birth weight and premature labor are common among WIMs [35], so our study highlights the need for gynaecological assessments, pregnancy tests and looking for signs of sexual abuse. Some studies [36] show that a high percentage of WIMs and girls have been victims of genital mutilation or they are at risk of it, and healthcare professionals can intervene to prevent and detect these risks during first emergency care.

WIMs arrive feeling exhausted from the journey by small boat, they have a history of vulnerability, and suffer physical and sexual abuse in the recruitment and travel-transit stages [5,37]. According to the United Nations Human Rights Council [38], it is important to guarantee the protection of women and female child migrants who have suffered traumas and sexual violence, guaranteeing adequate sexual and reproductive healthcare. The mother–child dyad is also important for detecting human trafficking. Our study shows that some children may be used for WIMs to gain access into Europe [39]. Some WIMs travel in small boats with babies who are not theirs [10]. Identifying these practices requires time, protocols and specific care [20,40]. According to Hendow [41], appropriate mechanisms need to be put into place to ensure proper identification of migrants, in particular, groups at risk. Human trafficking revolves around the mother–child dyad but it is difficult to get WIMs to collaborate as their children and families may be under threat [19]. Dealing with this delicate situation requires the collaboration of health care providers, cultural mediators, volunteers, non-governmental organisations (NGOs) and the police.

According to our findings, there is a need to improve communication, observe non-verbal language and to identify charms, provocative clothing, scars or tattoos [22,42]. Complex interventions are needed in order to effectively approach disease, barriers to medical attention and to restore dignity to WIMs and children [21]. Emergency care should give a more prominent role to cultural mediators [22]. A large part of emergency care to WIMs is carried out in police facilities, where WIMs are detained a maximum of 72 hours. Due to their history of violence, WIMs do not trust the police, which makes it more difficult to identify human trafficking [43,44]. In line with our results, other studies report the difficulty of caregiving in this environment due to a lack of coordination between healthcare personnel and police [45].

Consistent with our results, emergency care for WIMs must incorporate gynaecological assessment [14,22,25], mental healthcare provision [21], immunisation [12], information about the WIMs’ rights [41] and risk reports of victims of sexual violence and human trafficking [4]. There is a lack of studies that focus on emergency care for WIMs [46,47]. Our results identify vulnerability [37], conflicts between healthcare providers and the police [44], fear of deportation [48], a need for joint training and a lack of gender focus [23]. In addition, care is “not about simply rescuing them from dehydration, hypothermia and drowning, but sharing one’s humanity, and listening to their stories” [49]. Emergency care for WIMs who arrive by small boat should explore rape, sexual exploitation pregnancy, and human trafficking. The percentage of pregnant WIMs in our sample is higher than in other studies [50]; this may be due to the fact that many of them came to us from the Red Cross Humanitarian Settlement Centre, where they are given priority for care, as they make up such a vulnerable part of this population. 

Additionally, greater involvement on the part of receiving countries in expanding resources and comprehensive health interventions is needed [12,51]. National and European policies should be oriented towards the implementation of programmes to protect women and children irregular migrants [38]. As previously reported by Save the Children [34], it is difficult to collect data about this population because WIMs and children are afraid to share information, which in turn leads to a generalised lack of available evidence. Consequently, budget allocations and country involvement are still much needed in order to protect this vulnerable population at all stages of migration. One example of such budget allocation is the Asylum, Migration and Integration Fund [52], managed by the European Commission and its member states, which offers emergency aid in situations of migratory crisis. In addition, we would like to highlight the signing of bilateral agreements with transit countries, the creation of a Border Management Fund or the increase in material and human resources of the European Border and Coast Guard Agency (Frontex).

## 5. Limitations

The sample of WIMs in our study is small, as it is difficult to recruit participants due to their fear of speaking out. Interviews carried out with different WIMs could show contrasting results. A high percentage of WIMs in our study are pregnant, and, although their statements are similar to the rest, this may have an influence on the results. 

## 6. Conclusions

WIMs experience poverty, violence and inequality in their countries of origin, which motivates them to emigrate to Europe. Captured by the trafficking networks, they are victims of sexual exploitation and human trafficking during all phases of migratory travel. After arriving in Spain by small boat, WIMs require gynaecological assessment, pregnancy testing and screening for sexually-transmitted diseases and genital mutilation. By observing the mother-child relationship and the mothers’ behaviour, healthcare providers could identify violence and sexual exploitation. Emergency care puts safety before health and there is a lack of time, space, trained personnel and cultural mediators. From a gender perspective, institutions must develop policies and care protocols that are focused on WIMs who arrive by small boat.

## Figures and Tables

**Table 1:** Socio-demographic data of the participants (*N* = 23).

Participant	Sex	Age	Profession	Travel Status/Agency	Days (in Spain)	Country
WIM-1	Female	29	Immigrant	Pregnant	39	Nigeria
WIM-2	Female	34	Immigrant	Pregnant	32	Algeria
WIM-3	Female	19	Immigrant	Alone	42	Gambia
WIM-4	Female	18	Immigrant	Brother	56	Guinea Conakry
WIM-5	Female	33	Immigrant	Pregnant	45	Ivory Coast
WIM-6	Female	34	Immigrant	Alone	28	Nigeria
WIM-7	Female	30	Immigrant	Son, Pregnant	36	Morocco
WIM-8	Female	29	Immigrant	Alone	41	Senegal
WIM-9	Female	23	Immigrant	Alone	18	Gambia
WIM-10	Female	35	Immigrant	Daughter	16	Ivory Coast
WIM-11	Female	21	Immigrant	Husband and Son	19	Cameroon
WIM-12	Female	39	Immigrant	Pregnant	25	Nigeria
WIM-13	Female	28	Immigrant	Alone	31	Nigeria
KI-1	Female	50	Cultural mediator	Andalusian Government	-	Spain
KI-2	Male	49	Captain	Maritime Rescue	-	Spain
KI-3	Male	22	Team Leader	Psychologist		Spain
KI-4	Male	53	Captain	Maritime Rescue	-	Spain
KI-5	Male	47	Cultural mediator	Spanish Red Cross	-	Senegal
KI-6	Female	35	Cultural mediator	Spanish Red Cross	-	Morocco
KI-7	Female	29	Nurse	Andalusian Health Service	-	Spain
KI-8	Female	27	Nurse	Andalusian Health Service	-	Spain
KI-9	Female	25	Nurse	Andalusian Health Service	-	Spain
KI-10	Female	30	Nurse	Spanish Red Cross	-	Spain

WIM = woman irregular migrant, KI = key informant.

**Table 2:** Interview protocol.

Stage	Subject	Content/Example Questions
Introduction	Motives, reasons	Learn about the experiences and healthcare needs of WIMs during the migratory journey
	Objective	Improving first emergency care protocols for WIMs
Beginning	General introductory question	Can you tell us about your own particular experience, reasons for coming, and what you went through during the migratory journey?
Development	Conversation guide	Tell me about any problems that you faced from the time you left your country to when you arrived on the coast of Spain. Tell me about the rescue experience. What was your relationship with volunteers, nurses and police during maritime rescue and emergency care?
Closing	Final question	Do you have anything else you would like to share about your experiences during the migratory journey and emergency care?
	Appreciation	Thanking participants for their time and reminding them that their testimony will be very helpful.

WIMs = women irregular migrants.

**Table 3:** Themes, sub-themes and units of meaning.

Theme	Subtheme	Units of Meaning
The need for emergency care focused on WIMs.	WIMs as objects of sexual exploitation.	Specific needs, physical assessment, violence, marks and scars, sexual abuse, gynaecological assessment, abortion medication, genital mutilation, pregnancy test, signs of trafficking, psychosocial support.
	The mother–child dyad as the axis in human trafficking.	Awareness, active observation, identifying babies, collaboration amongst women, older children, network tools, young people.
Developing a gender policy for WIMs in emergency care.	Healthcare in a police-controlled setting: detecting weaknesses.	Lack of space, time and personnel, safety versus healthcare, fear and distrust, intimacy, communication, police custody, cultural mediator.
	Promoting screening and safety protocols focused on WIMs.	Gynaecological assessment, identifying sexually transmitted diseases, female genital mutilation, emotional instability, mental health, risk report, joint protocols, trafficking of women, identifying voodoo, cultural aspects, specific training.

WIMs = women irregular migrants.
